# Integrated Brain Metabolomics and Network Pharmacology Analysis to Reveal the Improvement Effect of Bai Chan Ting on Parkinson's Disease

**DOI:** 10.1155/2022/6113093

**Published:** 2022-12-06

**Authors:** Na Zhang, Jiaqi Fu, Xin Gao, Fang Lu, Yi Lu, Shumin Liu

**Affiliations:** ^1^Heilongjiang Drug Safety Evaluation Center, Heilongjiang, China; ^2^Institute of Traditional Chinese Medicine, Heilongjiang University of Chinese Medicine, Harbin, Heilongjiang, China

## Abstract

**Background:**

Baichanting (BCT),a traditional Chinese medicine prescription, is a combination of *Acanthopanax senticosus*, *Paeonia lactiflora*, and *Uncaria* three herbs, which has the effect of benefiting the kidney and calming the liver. The study was aimed at investigating the protective effect of BCT against Parkinson's disease using an integrated strategy of network pharmacology and brain metabolomics.

**Materials and Methods:**

By integrating network pharmacology with metabolomic research, the protective effect of BCT against PD was investigated using a transgenic mouse model for *α*-synuclein. The metabolite level and gene level components of BCT that might be anti-PD were separated out. Indicators of behavior and pharmacodynamics were employed to gauge the effectiveness of BCT on PD in the preliminary stages. Network pharmacology, which may be the target of BCT, screened the active substances and target genes. The use of metabolomics to identify potential biomarkers of PD, and then through network pharmacology and metabolic pathways to determine their regulatory enzymes and regulatory genes, improve the pathway mechanism of the disease, has important guiding significance for the in-depth study of the pathogenesis of PD.

**Results:**

101 putative target genes were identified by the network pharmacology analysis in relation to the treatment of PD with BCT. According to the functional enrichment analysis, the proposed mechanism was primarily related to the transport of neurotransmitters, the metabolism of arachidonic acid, dopamine, and alpha-amino acids, as well as the transport of dopamine and the negative regulation of amino acid transport. 25 distinct endogenous metabolites were shown to be potential biomarkers for the BCT for treating PD based on metabolomics. These metabolites were mostly implicated in the important methionine and cysteine, tyrosine, histidine, and arginine and proline metabolic pathways. These results somewhat agreed with those of the network pharmacology analysis.

**Conclusions:**

In conclusion, this study showed that BCT could delay the occurrence and development of PD by improving the brain metabolic disorder of *α*-Syn mice, which revealed the mechanism of BCT through multitarget and multipathway treatment, and provided a new explanation for the mechanism of anti-PD action. Our research, on the other hand, demonstrated that the network pharmacology-integrated metabolomics approach was a potent tool for discovering the active ingredients and mechanisms underlying the pharmacological effects of traditional Chinese medicine.

## 1. Introduction

Parkinson's disease (PD) is one of the common chronic neurodegenerative diseases, and its main symptoms are changes in motor function, such as bradykinesia, resting tremor, gait deformity, and postural instability, which eventually lead to the loss of motor ability and have become a major disease threatening human health [1]. Current drug treatment for PD mainly increases dopamine. Dopamine can antagonize central cholinergic neurotransmission, thereby alleviating its symptoms. At the same time there will be some toxic side effects, and can not effectively prevent the development of the disease. Subthalamic nucleus stimulation is used in clinical surgery to treat PD, but this method cannot avoid the sequelae of surgery; while nerve transplantation or gene therapy for PD is still in the preclinical research stage [2, 3]. The incidence of PD is increasing year by year and it is irreversible. The need for new strategies to effectively treat PD is extremely urgent. Therefore, researchers need to find new means or methods to carry out research on PD treatment. In the long-term practice of traditional Chinese medicine, the combination of disease and syndrome, the treatment of syndrome differentiation, and the addition and subtraction of prescriptions according to the syndrome have achieved good clinical effects. Therefore, further research on the mechanism of TCM in the treatment of PD has important clinical significance.

TCM theory holds that Parkinson's disease located in the brain. Its basic pathogenes is the imbalance of Yin and Yang in liver and kidney, syndrome of insufficient kidney essential to nourish the liver, “Syndrome of tremor” of movement disorder caused by asthenic yin causing predominant Yang. Modern physicians outline PD pathogenesis as Deficiency-excess mixing, liver-kidney deficiency, syndrome of asthenia of qi and blood as the primary aspect, and kidney failing to nourish liver, syndrome of liver wind stirring up internally as its secondary aspect [4]. Among them, nourishing kidney and tranquillizing liver yang therapy is the basic principle of treatment of PD in TCM clinical treatment [5]. Therefore, our laboratory proposes Baichanting Compounds (BCT), which is composed of *Acanthopanax senticosus* (Rupr. et Maxim.) Harms (AS), *Paeoniae Radix* Alba (PRA) and *Uncaria rhynchophylla* (Mia.) Miq. Ex Havil (UR) based on Chinese medicine for suppressing hyperactive liver and benefiting kidney treatment to treat PD. This formula has won a national patent (ZL 201110260536.3). The compound gets the effects of nourishing liver and kidney, suppressing hyperactive liver for lowering adverse qi, benefiting qi for promoting production of blood, calming wind, and dredging collateral. AS is good at tonifying qi and spleen, tonifying kidney and tranquilization liver as primal medicine, can protect neurons by inhibiting the damage of 1-methyl-4-phenylpyridine to nerve cells [6]. PRA can replenish blood and nourishing liver, suppressing hyperactive liver, and subsiding yang as assistant drugs, which are beneficial for primal medicine to play nourishing liver and kidney. It can also significantly antagonize the ruin of nigrostriatal neurons in PD models, and significantly improve the symptoms of motor retardation [7]. UR has the curative effect of calming liver wind, which can not only help PRA to exert better medicinal effect, so it is adjuvant and can produce good protection against 6-hydroxydopamine-induced cytotoxicity [8]. The combination of the three drugs can achieve the curative effects of invigorating kidney and liver, benefiting qi for activating blood circulation, relieving spasm by calming endogenous wind. The combination of three drugs in the recipe can reduce the levels of IL-1*β*, IL-6, TNF-*α,* and other inflammatory proteins, and improve the neuroinflammatory response [9]. It treats PD with its features of multicomponent, multitarget, multiefficacy, and low toxicity.

Metabolomics is an emerging technology developed after genomics and proteomics in recent years to identify and analyze endogenous small molecular substances and their dynamic changes in biological organisms. It is mainly to study the changes of endogenous metabolites produced by specific organisms after intervention, and to qualitatively and quantitatively analyze the key compounds in the metabolic cycle, so as to clarify the composition of all components and their interrelationships under specific conditions [10]. Therefore, metabolomics can simultaneously and systematically mine changed molecules or their associated molecular regulatory networks. Metabolomics takes the study of the overall metabolic changes of the organism as the starting point, comprehensively reflects the metabolic changes of various tissues in the organism caused by the action of Chinese medicine, and then clarifies the mechanism of action of Chinese medicine, which is in line with the characteristics of the integrity of Chinese medicine [11]. Syndrome differentiation and treatment are the characteristics and essence of TCM. As a key technology and research method of systems biology, metabolomics can dynamically reflect the state of the body and conform to the holistic principle of syndrome differentiation and treatment and the effect of compound prescriptions in TCM. Metabolomics elucidates the occurrence and development mechanism of disease and the related mechanism of drug treatment by exploring the changes of endogenous metabolites. It is increasingly widely used in the study of TCM syndromes and compound prescriptions. In recent years, it has been increasingly used in the study of the mechanism of action of TCM, and the rise of network pharmacology has provided the possibility for the screening of active components of traditional Chinese medicine and the research on the mechanism of action of multiple targets [12]. Network pharmacology research uses a variety of databases to screen out known drug components and target information about specific diseases, and uses various software to build a “disease-gene-target-drug” multidimensional network model, and finds it through data mining and network analysis. Key nodes in the network, and finally comprehensively analyzes and study the regulatory effect of drugs on related signaling pathways, and then predict pharmacodynamic active components, in vivo targets, and mechanisms of action. Network theory is especially suitable for exploring the relationship between a large number of biological data. Metabolomics can screen endogenous or exogenous metabolites, and the combination of the two can provide new research ideas for the systematic study of drug-disease mechanism, and reveal the complex relationship between multiple metabolites and multiple targets. Therefore, finding the active components of network pharmacology and confirming the effective active products of metabolic processes are the first steps of integrated analysis, and then appropriate research methods can be adopted according to different research purposes.

The purpose of this study was to choose *α*-synuclein (*α*-Syn) transgenic mice for the experiments and employ metabolomics and network pharmacology to investigate the mechanism of BCT in the treatment of PD. By comparing the changes of metabolites in vivo, we can find the regulatory pathways and biomolecular networks related to these products. And just try to sort out its potential anti-PD components from the metabolic and molecular levels, explore its core targets and key pathways, explain its metabolic regulation mode, and explore its potential anti-PD mechanism. This has a significant impact on how the scientific importance of BCT treatment for PD is understood. A method for comprehending the underlying mechanisms in PD and identifying possible BCT targets for the therapy of the condition is the combination of metabolomic and network pharmacology studies.

### 1.1. Chemicals and Reagents

*Acanthopanax senticosus*, *Radix Paeoniae Alba*, and *Uncaria rhynchophylla* were purchased from Tongrentang Chinese Medicine (Beijing, China). Sodium carboxymethyl cellulose (CMC-Na) was purchased from Foshan Rite Chemical Co., Ltd.; HPLC-grade methanol, acetonitrile, and formic acid were purchased from Fisher Scientific (Fair Lawn, NJ, USA). Nitric oxide (NO), Superoxide Dismutase (SOD), Malonic dialdehyde (MDA), and Dopamine (DA) were purchased from the Nanjing Jiancheng Bioengineering Institute (Nanjing,China).

Preparation of BCT Chinese herbal formula: the optimal compatibility ratio of each component in BCT used in our experiment has been obtained from the previous experiments of the research group, the best compatibility ratio is “*Acanthopanax senticosus*: *Paeoniae Radix Alba*: *Uncaria rhynchophylla* = 54.0: 45.0: 82.5”. The medicinal materials of AS were extracted with 70% ethanol under reflux, and after concentration, they were separated and purified by macroporous resin, and the eluted part with 30% ethanol was collected, and then freeze-dried after concentration. The medicinal materials of PRA were extracted by refluxing with 90% ethanol, and then separated and purified by macroporous resin after concentration. The medicinal materials of UR were extracted with 90% ethanol, and after concentration, they were separated and purified by macroporous resin, and the eluted part of 80% ethanol was collected, concentrated and freeze-dried. The above components are prepared by dissolving an appropriate amount of powder in a 0.5% CMC-Na aqueous solution.

### 1.2. Animal Treatment

A total of 20 six-month-old male WT *α*-synuclein transgenic mice and 10 matched male C57BL/6 mice included in the study were purchased from Jiangsu Jicui Yaokang Laboratory Animal Technology Co., Ltd. (Nanjing, China) at 6 month (SCXK (su) 2018-0008). The mice were housed individually in wire cages under controlled humidity (40-50%) and temperature (22 ± 1°C) with a light-dark cycle of 12 h. All mice had free access to fodder and drinking water throughout the study period and were allowed at least one week to adapt to the laboratory environment before experiments. The protocol was approved by the Committee on the Ethics of Animal Experiments of Heilongjiang University of Chinese Medicine, China, and the approval number was 2018052325.

After acclimatization, 20 *α*-synuclein transgenic mice were randomly divided into two groups such as the *α*-synuclein group and *α*-synuclein + BCT treated group (363.00 mg/kg-1). The matched male C57BL/6 mice were selected as the control group and administered with saline (20 ml/kg daily). The optimal dose obtained from the previous experiments of the research group [13]. The administration volume was 50 ml/kg, and it was administered by gavage for 20 consecutive days.

### 1.3. Behavioral Experiments

Climbing pole experiment: choose a wooden pole with a length of 100 cm and a thickness of 1.5 cm, and wrap the whole pole with tape to prevent the mice from slipping during the pole climbing process. The tail of the mouse holding the rod puts its head down on the top of the rod and allows it to climb down naturally, from the two hind limbs standing on the top of the rod to the two forelimbs touching the bottom platform of the rod. The time for the mouse to climb the rod is required for this process. The total time was recorded with a stopwatch; each mouse was recorded three times, and the average was taken.

Autonomic activity experiment: using a mouse autonomic activity tester, the animals were placed in the separation reaction box for 5 minutes, and the number of autonomous activities of the mice within 5 minutes was recorded. The number of voluntary activities is the sum of the number of vertical and horizontal movements.

### 1.4. Biochemical Parameters

On the 21st day, brain tissue samples were grounded, and the supernatant was taken after homogenization. The expressions of SOD, MDA, NO, and DA in brain tissue were detected by the assay kit methods.

### 1.5. Sample Collection and Preparation

After the last administration, mice were sacrificed by decapitation, and whole brain tissue were immediately removed and snap-frozen in liquid nitrogen. The brain samples were divided into two aliquots, brain was obtained from one aliquot by centrifugation at 3500 rpm for 15 min after the brain samples clotted, then analyzed using a reagent test kit to test for MDA, DA, SOD, and NO. The obtained brain was transferred into Eppendorf tubes and stored at 80°C for metabonomics analysis. After the samples were slowly thawed at 4°C, an appropriate amount of samples was taken and added to precooled methanol/acetonitrile/water solution (2 : 2 : 1, v/v), mixed by vortex, sonicated at low temperature for 30 min, stood at -20°C for 10 min, 14000 centrifuge at g 4°C for 20 min, took the supernatant and dry it in vacuo, add 100 *μ*L of acetonitrile aqueous solution (acetonitrile: water = 1 : 1, v/v) to reconstitute, vortex, and centrifuge at 14,000 g for 15 min at 4°C, removed the supernatant injection analysis. At the same time, the quality control (QC) sample was prepared by blending equal volumes from each of the 30 samples and was analyzed in every 10 brain samples for evaluating the stability and performance of the system.

### 1.6. Chromatography

Utilizing the UHPLC, chromatographic separation was carried out (1290 Infinity LC, Agilent Technologies). Samples were examined using a 2.1 mm 100 mm ACQUIY UPLC BEH 1.7 m column for HILIC separation (Waters, Ireland). The mobile phase contained A = 25 mM ammonium acetate and 25 mM ammonium hydroxide in water and B = acetonitrile in both ESI positive and negative modes. The gradient profile used for the separation was a linear gradient of 85% B for 1 min, 85–65% B for 1–11 min, 65–40% B for 11–11.1 min, 40% B for 11.1-15.1 min, and 40-85% B for 15.1–15.2 min, with a 5 min reequilibration period employed. A 2.1 mm 100 mm ACQUIY UPLC HSS T3 1.8 m column (Waters, Ireland) was utilized for RPLC separation. In ESI positive mode, the mobile phase contained A = water with 0.1%formic acid and B = acetonitrile with 0.1%formic acid; and in ESI negative mode, the mobile phase contained A = 0.5 mM ammonium fluoride in water and B = acetonitrile. The metabolites were eluted using a linear gradient of 1% solvent B for 1.5 min, 1-99% solvent B for 1.5-11.5 min, 99% solvent B for 3.5 min, 99%-1% solvent B for 11.5-11.6 min, and 99% solvent B for 11.6-15 min. The column temperatures were maintained at 25°C and the gradients flowed at a rate of 0.3 mL·min^−1^. Each sample was given a 2 *μ*L aliquot injection.

### 1.7. Mass Spectrometry

AB Sciex TripleTOF 6600 (Shanghai Applied Protein Technology Co., Ltd.) mass spectrometry was performed using ESI in both positive and negative modes. The following conditions were set for the ESI source: source temperature: 600°C, IonSpray Voltage Floating (ISVF): 5500 V, Ion Source Gas1 (Gas1) as 60, Ion Source Gas2 (Gas2) as 60, curtain gas (CUR) as 30. The device was configured for MS-only acquisition with an m/z range of 60–1000 Da and a TOF MS scan accumulation time of 0.20 s/spectra. The apparatus was set up for automatic MS/MS acquisition with the m/z range of 25–1000 Da and the accumulation time for product ion scan at 0.05 s/spectra. Information dependent acquisition (IDA) is used to acquire the product ion scan, and the high sensitivity option is chosen. The following criteria were established: Declustering potential (DP), 60 V (+) and 60 V (−); exclude isotopes within 4 Da; candidate ions to monitor every cycle: 10. The collision energy (CE) was fixed at 35 V with 15 eV;

### 1.8. Data Processing and Biomarker Selection

After normalized to total peak intensity. Combined with Progenesis QI software and EZinfo2.0 software (software in MarkerLynx1.4 workstation), brain tissue analysis was performed, principal components analysis (PCA) was used for unsupervised statistical analysis, and partial least-squares discriminant analysis (PLS-DA) was used to analyze the brain tissue. The test samples were analyzed and the corresponding score chart was obtained.

The SIMCA-P software 12.0 should be used for the default cross-validation and 200 random permutations should be utilized to test the data in order to prevent overfitting of the PLS-DA model. Each variable in the PLS-DA model had its variable importance in the projection (VIP) value calculated to show how it contributed to classification. To further assess each metabolite's importance, the VIP value > 1 was used to a Student's *t*-test at the univariate level. *P* values less than 0.05 were regarded as statistically significant. Primary identification of the metabolites was conducted in QI by searching the Human Metabolome Database (HMDB), Kyoto Encyclopedia of Genes and Genomes (KEGG) and MetaboAnalyst (http://www.metaboanalyst.ca/) for related metabolic pathway enrichment analysis and network construction are performed to provide more biological information on the physiological and pathological states of differential metabolic markers, and to find differential metabolites and metabolic pathways related to the study of diseases.

### 1.9. Network Pharmacology Analysis

#### 1.9.1. Composite Compounds and Target Genes of BCT

The chemical information (*Paeoniae Radix Alba* and *Uncaria rhynchophylla*) were found using the TCMSP (https://old.tcmsp-e.com/tcmsp.php. updated in May 2014) databases. The compounds with OB ≥30% and DL ≥0.18 were chosen for the ensuing analyses. The main active components of *Acanthopanax senticosus* are phenolic glycosides. The glycosides with higher content in the total glycosides of *Acanthopanax senticosus* were selected through literature search, and the PubChem database was used (https://pubchem.ncbi.nlm.nih.gov/) to retrieve the simplified molecular-input line-entry system (SMILES) for each component. Enter the SMILES formula, import the SwissTargetPrediction (http://www.swisstargetprediction.ch/) platform, and obtain the prediction results of the potential targets of the selected components. Through a literature research, more details about the compounds were discovered.

#### 1.9.2. Prediction of Potential Targets of BCT Acting on PD

Using “Parkinson's Disease” as the key word, the GeneCards, OMIM, and DrugBank databases were searched, and related genes were obtained. The obtained drug targets and Parkinson's disease genes are mapped and intersected to obtain potential targets of BCT in the treatment of PD. Using the Protein Database's UniProt Knowledgebase search function (http://www.uniprot.org/), all of the obtained targets were changed to their official symbols.

#### 1.9.3. Pathways Enrichment Analysis and Network Construction

To explore the combination mechanisms of BCT for PD, the pathways enrichment was performed using the servers *viz* DAVID Bioinformatics Resources 6.8. The pathways with 0.05 *P* values were selected. Meanwhile, the acquired target genes were submitted to the STRING (http://string-db.org/) database to obtain a protein-protein interaction network. Furthermore, imported into Cytoscape 3.8.0 software, set the parameters so that the node size and color depth reflect the value of the degree, the thickness of the edge reflects the combination rate score, and establish a PPI network diagram.

### 1.10. Experimental Validation

By using quantitative real-time PCR, the hub targets that connected systematic pharmacology and metabonomics above were confirmed (qPCR). Following the manufacturer's directions, total RNA was isolated from brain using TRIzol Reagent (Thermo 230 Fisher Scientific). As directed by the Primescript RT reagent Kit, the RNA was reverse transcribed into cDNA (Dalian Bio-Bio Co., Ltd., China). In Table 1, the primer sequences are displayed. The SYBR Premix Ex Tap™ kit (TaKaRa business) is utilized in the RT-PCR reaction process, and GAPDH is employed as an internal control. The relative expression levels of mRNA were estimated by the 2^-*ΔΔ*CT^ method, and the studies were performed three times in each group using the expression levels of ARG1, P4HA1, NOS3, NOS1, and ALDH2 in brain tissue as the control.

### 1.11. Statistical Analysis

The Student's *t*-test was further applied to assess the significant difference of these features between group using SPSS (version 21.0; Beijing Stats Data Mining Co. Ltd., China). A biomarker was chosen when a value of *P* < 0.05 was deemed statistically significant. The effect of BCT for PD was demonstrated by comparing the trend of the intensity of these biomarkers between the BCT-treated group and the model group.

## 2. Results

### 2.1. Behavioral Indicators

**Figure 1:**
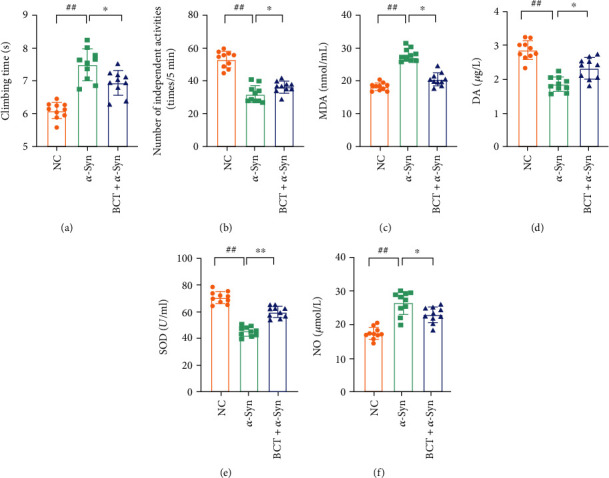
The effect of BCT on the brain tissue indexes of *α*-Syn mice. (a), Pole climbing test results; (b), autonomic activity experiment results; (c), MDA content results; (d), DA content results; (e), SOD content results; (f), NO content results. Values expressed as mean ± SD (*n* = 10). ^#^ Significantly different from the control group at *P* < 0.05. ^##^ Significantly different from the control group at *P* < 0.01. ^∗^ Significantly different from the model group at *P* < 0.05. ^∗∗^Significantly different from the model group at *P* < 0.01 (one-way ANCOVA).

As shown in Figures 1(a) and 1(b). The pole climbing test is the most commonly used behavioral detection method for PD animal models, and it has a good detection effect on the coordinated movement ability of animals. Compared with the control group, the climbing time of *α*-Syn transgenic mice was significantly increased (*P* < 0.05); compared with the model group, the climbing time of each BCT administration group was significantly decreased (*P* < 0.05). In addition, it was also found that *α*-Syn mice also showed a decrease in the number of autonomous activities. After administration of BCT, the number of autonomous activities in the high-dose group increased significantly (*P* < 0.05), indicating that BCT can significantly improve the motor dysfunction of PD mice.

### 2.2. Biochemical Indices

As shown in Figures 1(c)–1(f), the experimental results showed that the activity levels of NO, DA, SOD, and MDA in the brain tissue of the transgenic model mice were significantly changed compared with those of the normal mice. Among them, the content of NO in the *α*-Syn group was significantly increased (*P* < 0.01), and the content of DA was decreased (*P* < 0.05). The progressive decline of DA in the striatum after PD is the main cause of motor symptoms. It was also found that MDA levels in *α*-Syn mice were significantly increased compared with normal mice, while SOD levels were significantly reduced, indicating that the nerve cells of PD mice were damaged by oxidative stress. After administration of BCT, it can increase the content of SOD in the brain tissue, reduce the level of NO, and improve the state of oxidative stress, thereby protecting the brain.

### 2.3. Metabolic Profiling

**Figure 2:**
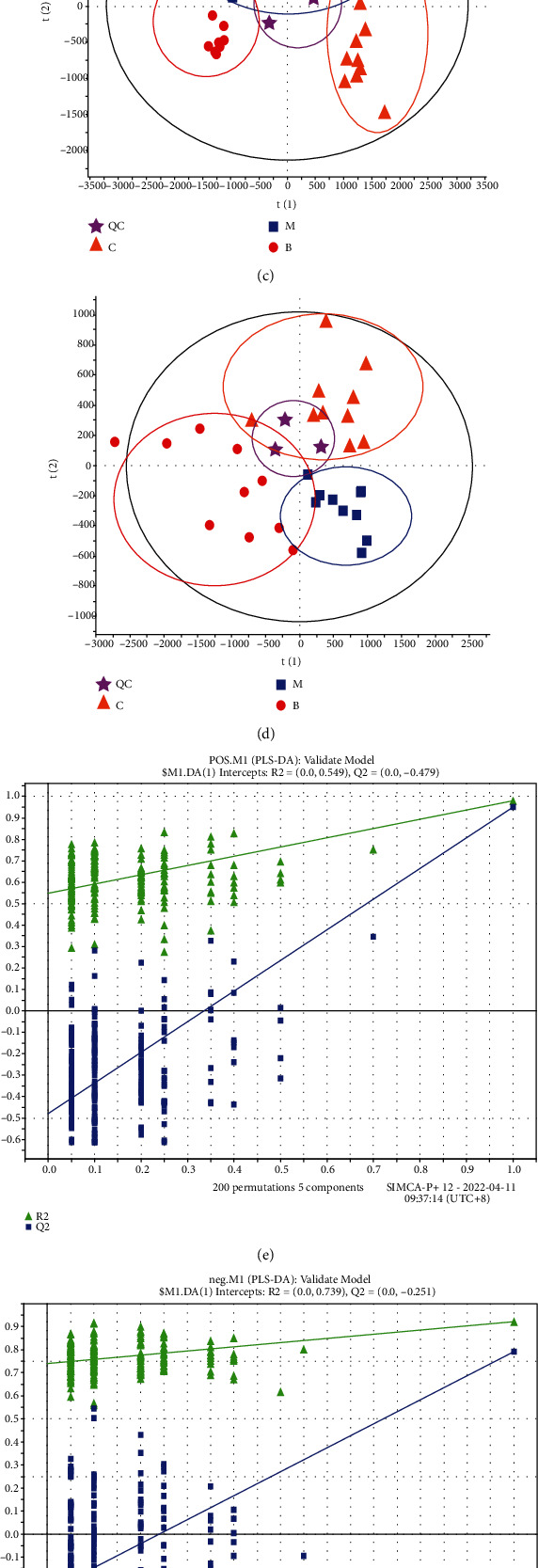
Multivariate statistical analysis of metabolic profiles of brain tissue in *α*-Syn mice. PCA score map of positive ion mode (a) and negative ion mode (b); PLS-DA score map of positive ion mode (c) and negative ion mode (d) PLS-DA model verification diagram of positive ion mode (e) and negative ion mode (f); VIP-Plot plots of positive ion mode (g) and negative ion mode (h).

The brain tissue samples of mice in each group were detected in positive/negative ion mode by UHPLC. In order to further study the changes of metabolites in brain tissue, two pattern recognition techniques, namely PCA and PLS-DA, were used for metabolic data analysis. The brain tissue PCA score map with quality control samples is shown in Figures 2(a) and 2(b), in which the quality control samples (QC) showed relatively tight aggregation. After obtaining the score graph of PCA after standardized treatment (Figures 2(a) and 2(b)), it can be seen that the data points in the figure are closely clustered. In addition to the observation that the data points have partial overlap, the data of each group are significantly clustered, indicating that the metabolites have been significantly changed. PLS-DA assays were subsequently performed to show the differences between groups, as shown in Figures 2(c) and 2(d). In the PLS-DA score map in the positive/negative ion mode of brain tissue, it was observed that the data points of the control group, the model group and the BCT group all presented their own clusters, which were located in 3 different areas of the scatter score map. In order to avoid model overfitting in the modeling process and ensure the validity of the model, we used SIMCA-P software to perform cross-validation and permutation testing. As shown in Figures 2(e) and 2(f), the parameters of the PLS-DA model, including the R_2_Y and Q^2^ values (0.959 and 0.877 for the positive mode, 0.945 and 0.812 for the negative mode), are all >0.5, and the Q^2^ regression line intercept is negative, and the blue Q^2^ value on the left and the green *R*^2^ value are both smaller than the value on the right. It can be seen that the positive and negative ion PLS-DA model has a low risk of overfitting and has good predictive ability.

The variable importance in projection (VIP) of the derived multivariate analysis of the PLS-DA model might preliminary screen out the metabolites that differ across the groups based on the PLS-DA results (Figures 2(g) and 2(h)). To further weed out differential metabolites, the *P* value and fold change (FC) value of univariate analysis can be combined. Combined with the *P* value of *t*-test of univariate analysis, set the threshold *P* value <0.05 for differential metabolite screening. According to the method of data processing and metabolite identification, VIP ≥1 in PLS-DA mode, FC>1.2 or FC<0.8, using analysis of variance (P < 0.05) as a screening criterion to explore different metabolites associated with Parkinson's disease. A total of 25 metabolites (12 in positive mode and 13 in negative mode) were identified based on secondary spectral information and the HMDB database. The retention time, detection value, standard value, molecular formula, ion pattern, and metabolite name of these biomarkers are shown in Table 2.

**Figure 3:**
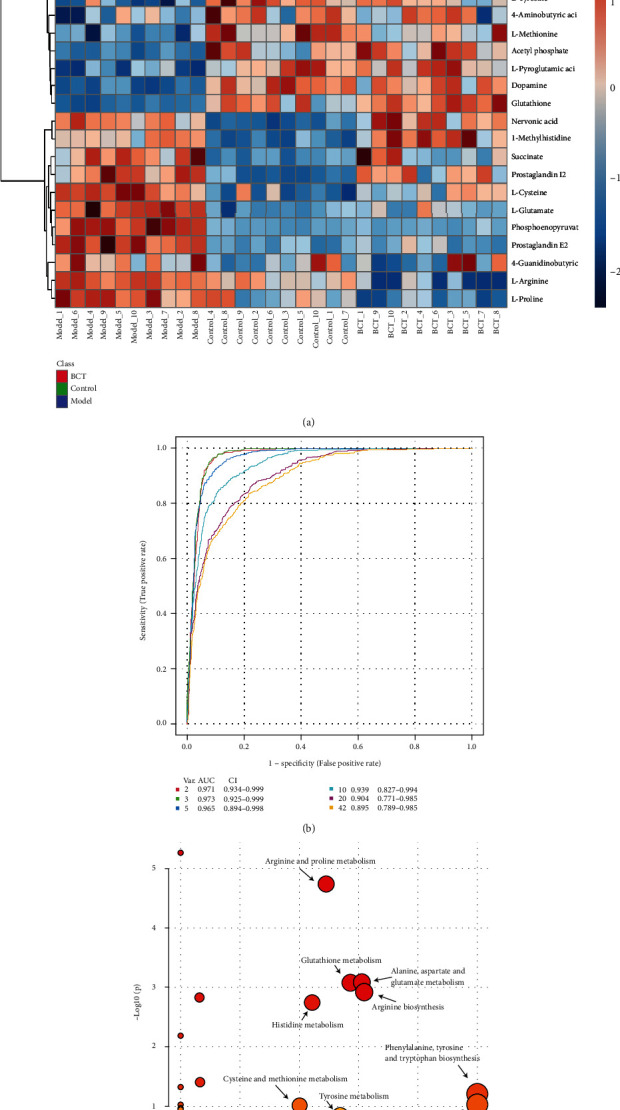
Brain tissue metabolites and metabolic pathways. (a). Cluster analysis heat map; (b). metabolite ROC curve analysis; (c). analysis map of metabolic pathways.

Changes in the content of 25 potential biomarkers between groups were visualized from the heat map (Figure 3(a)). The potential biomarker levels of the normal group and the PD model group could be clearly distinguished, and the potential biomarker levels of the BCT group and the PD group could also be clearly distinguished. The content levels of potential markers in the BCT group were closer to those in the control group than in the model group.

To evaluate the sensitivity and specificity of metabolites, we performed ROC curve analysis. As shown in Figure 3(b), by comparing AUCs, the predictive power of potential metabolites was determined. All AUC values for metabolites were>0.8 at 95% confidence intervals, and these results showed improved sensitivity and specificity for all identified potential biomarkers.

Using databases such as HMDB and KEGG, the structural information of the 25 potential markers screened out was determined, and imported into MetaboAnalyst 5.0 to analyze metabolic pathways online. Pathways with this value were considered as potential target pathways. As shown in Figure 3(c). The metabolic disorder in the brain tissue of PD mice is mainly related to amino acid metabolism.

### 2.4. Network Pharmacology Analysis

**Figure 4:**
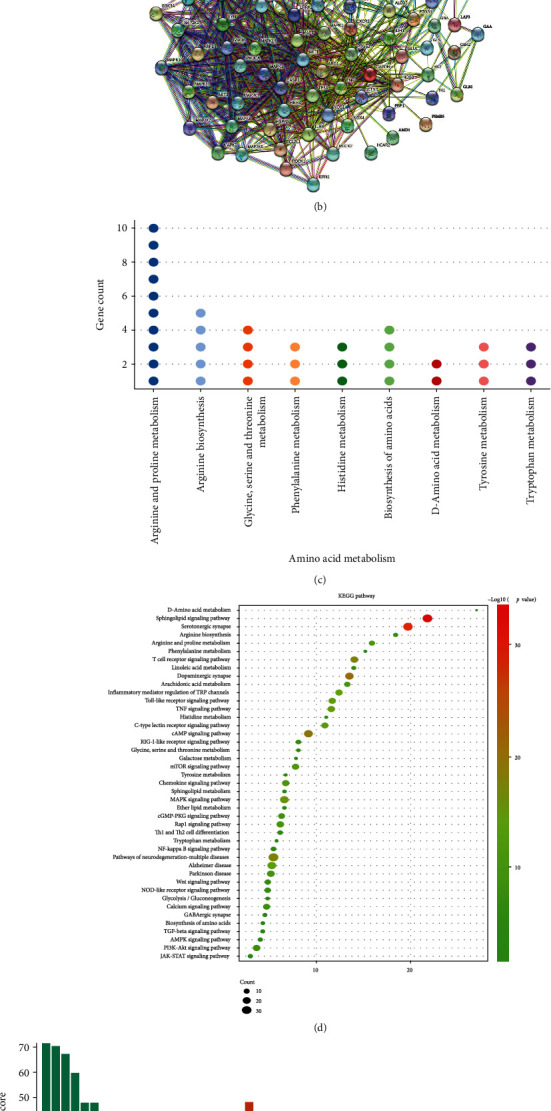
Network pharmacology analysis. (a). BCT anti-PD “component-target-disease” network diagram; (b). protein-protein interaction network diagram; (c). amino acid metabolism-related target relationship diagram; (d). KEGG enrichment analysis; (e). GO enrichment analysis show.

According to the screening criteria, the related active compounds of *Acanthopanax senticosus*, *Paeonia lactiflora*, and *Uncaria* were obtained by searching the TCMSP database and literature, and the duplicate targets were deleted, and a total of 362 active ingredient-related targets were obtained. A total of 4028 Parkinson's related targets were obtained by searching the related disease gene database. The potential targets of fibrillation were intersected with the above-mentioned Parkinson's disease targets, and 101 intersecting targets were obtained (Table 3). Cytoscape v3.8.2 was used to display the network relationship diagram of the anti-PD “component-target-disease” of fibrillation (Figure 4(a)).

Cytoscape 3.8.2 was used for visual analysis to construct a protein-protein interaction network (Figure 4(b)). Based on the results of metabolomic analysis, we found a correlation between BCT treatment of PD and amino acid metabolism. Potential targets involved in amino acid metabolism were identified from the intersection targets, and a relationship map of amino acid metabolism-related targets was constructed (Figure 4(c)).

The resulting BCT anti-PD potential target genes were subjected to GO annotation and KEGG pathway enrichment analysis by DAVID (Figures 4(d) and 4(e)). GO enrichment analysis showed that there were mainly modulation of synaptic transmission, protein tyrosine kinase activity, neurotransmitter transport, arachidonic acid metabolic process, dopamine metabolic process, glial cell differentiation, alpha-amino acid metabolic process, dopamine transport, and negative regulation of amino acid transport. According to the KEGG enrichment analysis, the significantly affected pathways were sphingolipid signaling pathway, serotonergic synapse, dopaminergic synapse, arginine and proline metabolism, GABAergic synapse, biosynthesis of amino acids, D-Amino acid metabolism, arginine biosynthesis, histidine metabolism, tyrosine metabolism, and tryptophan metabolism.

### 2.5. Comprehensive Analysis of Metabolomics and Network Pharmacology

**Figure 5:**
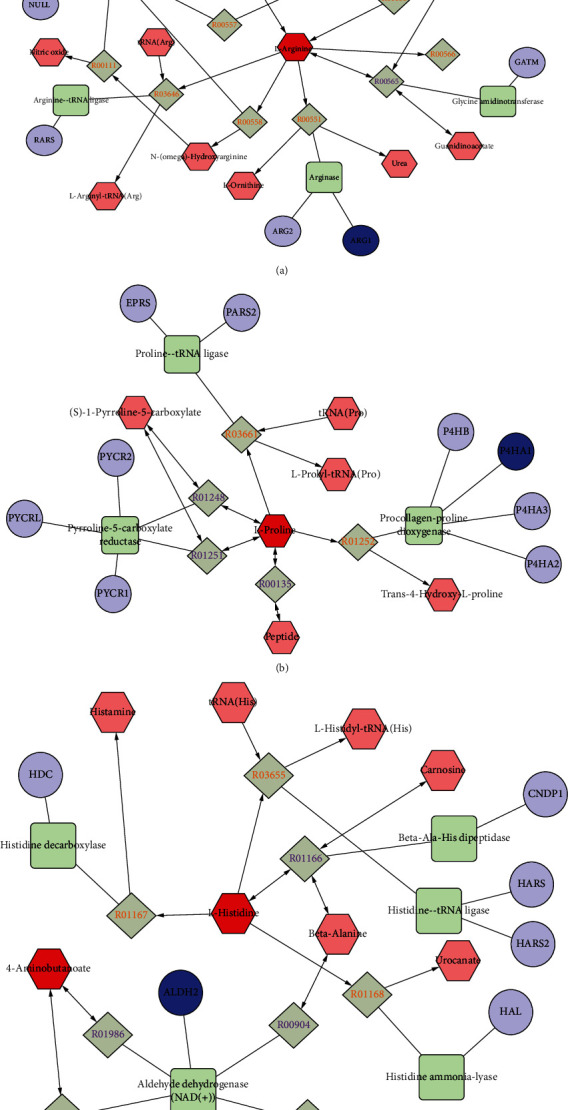
Compound-reaction-enzyme-gene network of key metabolites and targets. Red hexagons, gray diamonds, green round rectangles, and purple circles represent active compounds, reactions, proteins, and genes, respectively.

To comprehensively understand the mechanism of BCT against PD, we constructed an interaction network based on metabolomics and network pharmacology. The differential metabolites were imported into the MetScape plugin of Cytoscape to collect compound-reaction-enzyme-gene networks (Figure 5). MetScape analysis revealed that five hub genes in network pharmacology (ARG1, P4HA1, NOS3, NOS1, and ALDH2) were associated with metabolites in metabolomics. The related key metabolites are L-Histidine, L-Arginine, L-Proline, 4-Aminobutyric acid, 1-Methylhistidine, and argininosuccinic acid. As shown in Table 4. These genes and metabolites affect the same metabolic pathways (arginine and proline metabolism and histidine metabolism). They may play an important role in the therapeutic effect of BCT on PD.

## 3. Discussion

Parkinson's disease is a common central nervous system disease. The main pathological features of PD are decreased striatal DA levels, irreversible and selective loss of dopamine neurons in the substantia nigra, and the appearance of Lewy bodies in the cytoplasm of substantial nigra neurons. In this study, *α*-synuclein transgenic C57BL/6 mice were innovatively selected as experimental subjects. *α*-Synuclein is a presynaptic protein, which is the main component of Lewy bodies and Lewy neurites in PD characteristic lesions. This will make the mice show PD symptoms such as progressive loss of motor coordination and accurately replicate the PD model. Choosing this breed of mice as the experimental object can more accurately study the abnormal brain function of PD, such as appearance behavior and changes in brain dopamine content. BCT is simplified from the clinical treatment experience of PD, Fang Fuyuan Ping channing. It is a traditional Chinese medicine compound that can repress hyperactive liver Yang, nourish Qi and blood, and expelling wind and relieving convulsion. In this study, *α*-Syn transgenic animal model was used to conduct experiments, and metabolomics combined with network pharmacology technology was used to reveal the neuroprotective effect and mechanism of BCT from multiple pathways, multiple targets, and multiple levels, laying a foundation for independent research and development of TCM compounds that can treat or assist PD. The research group measured the oxidative stress-related indicators in the substantia nigra of mice in the early stage to study the relationship between BCT, PD model and oxidative stress response. The results showed that the cells in the substantia nigra of *α*-syn mice exhibited typical apoptotic morphological changes, such as cells becoming smaller, rounder, and nuclear pyknosis, and the apoptosis rate was significantly increased. After BCT intervention, the levels of SOD and GSH-Px were significantly increased, while the levels of MDA and MAO-B were significantly decreased. MAO-B decomposes 70% of dopamine in the human brain. By inhibiting the activity of MAO-B, it can inhibit its effect on dopaminergic nerves. The purpose of the degradability of the element, indicating that BCT has a significant protective effect on nerve cells [14, 15]. Our study once again validated the therapeutic potential of BCT in *α*-Syn mice by improving motor coordination and inhibiting oxidative stress in brain tissues. Our results on the effect of BCT are broadly consistent with that of previous studies. Network pharmacology analysis showed that multiple components of BCT exert therapeutic effects on PD by modulating multiple targets and pathways. Among these components, major compounds of BCT have been shown to have therapeutic potential for PD, confirming the therapeutic value of BCT in this disease [16–18]. Metabolic pathway analysis showed that BCT could regulate 25 PD-related metabolites, of which 12 metabolites (L-histidine, L-arginine, and L-proline) were related to amino acids. In addition, six amino acid metabolism pathways were predicted to be related to PD treatment through the BCT network pharmacology, including arginine and proline metabolism, histidine metabolism, glycine, serine, and threonine metabolism, tyrosine metabolism, and tryptophan metabolism. Exploring the relationship between multiple ligands and multiple targets through network pharmacology analysis is of great significance for the development of novel adjuvant drugs.

Amino acids play an important role in the differentiation, growth, and development of neurons, including increasing synaptic plasticity and terminal density, promoting the growth of axons and dendrites, reducing the loss of protrudation, enhancing the activity of the brain nervous system, maintaining the normal physiological function of the brain, and delaying its atrophy and degeneration [19–21]. When peroxidation occurs in the body due to ischemia, hypoxia, or tissue damage, a large number of free radicals will be generated in the body, resulting in damage to nerve cells. Amino acids in cerebrospinal fluid can be absorbed by neurons, inhibit the generation of free radicals in the brain, remove reactive oxygen species, and protect neurons [22].

The differential metabolites of 1-methylhistidine and L-histidine were selected to participate in histidine metabolism. Histidine is an amino acid containing imidazole ring with unique physiological characteristics. It is the biosynthetic precursor of several hormones (such as thyrotropin-releasing hormone) and the neurotransmitter histamine, as well as the principal constituent amino acid of body proteins and some functional proteins (such as histone and hemoglobin). Histidine can regulate intracellular pH and nervous system, and play important anti-inflammatory, antisecretion, antioxidation, and other functions in vivo. The lack of histidine and the deficiency of histidine metabolism causes a heavy blow to the various systems of the body [23]. Histidine is involved in nervous system regulation mainly through the action of histamine, which is synthesized by histidine decarboxylase. Histamine is generated by a variety of immune cells and released from basophils and mast cells to increase vascular permeability of capillaries and tail veins, causing inflammatory reactions and allergic symptoms in the immune system of organisms. At the same time, as a neurotransmitter in the brain, it is linked to a variety of central nervous system diseases [24]. Studies have shown that histamine plays an important part in brain development and nerve regeneration by activating the histaminergic nervous system, which in turn activates the H1 and H4 receptors on neural stem cells and promotes the regeneration of injured nerve fibers [25, 26]. Compared with the control group, L-histidine content in *α*-Syn transgenic mice decreased significantly, and the 1-methylhistidine content increased. When organisms are deficient in histidine, globin synthesis is reduced to reduce histidine consumption and increase histidine utilization, leading to a rise in histamine index levels in a short time. After BCT intervention, L-histidine content increased significantly, while 1-methylhistidine content did not change significantly. It was speculated that BCT could make its imidazole ring play a role in clearing excess ROS produced by brain tissue, maintaining antioxidant system and mitochondrial function, and retaining high-energy phosphoric acid by increasing L-histidine. Inhibition of cytokines and growth factors associated with cell and tissue damage to maintain cell fluid homeostasis and ameliorate oxidative stress damage.

Tyrosine is synthesized from phenylalanine in the dopaminergic neurons of the brain, and under the action of amino acid decarboxylase, tyrosine is generated, and tyrosine is further metabolized into neurotransmitters and hormones such as dopamine, norepinephrine, and epinephrine. Accumulation or deficiency of tyrosine in the body can lead to abnormal changes in growth and development, especially in stress states that require more tyrosine supplementation, and these changes caused by tyrosine toxicity are associated with oxidative stress in the brain, astrocytes, and ultimately cognitive impairment [27, 28]. The level of tyrosine in *α*-Syn transgenic mice is reduced, which may be due to PD making phenylalanine too high, inhibiting tyrosine hydroxylase activity, reducing tyrosine content, and hindering normal neurotransmitters (dopamine and normethyl), adrenaline synthesis, so the abnormal tyrosine metabolism will cause the disorder of phenylalanine metabolism and energy metabolism, which will affect the overall condition of the body. After BCT intervention, the levels of tyrosine and its metabolite dopamine were upregulated, indicating that BCT can treat PD by regulating tyrosine metabolism.

**Figure 6:**
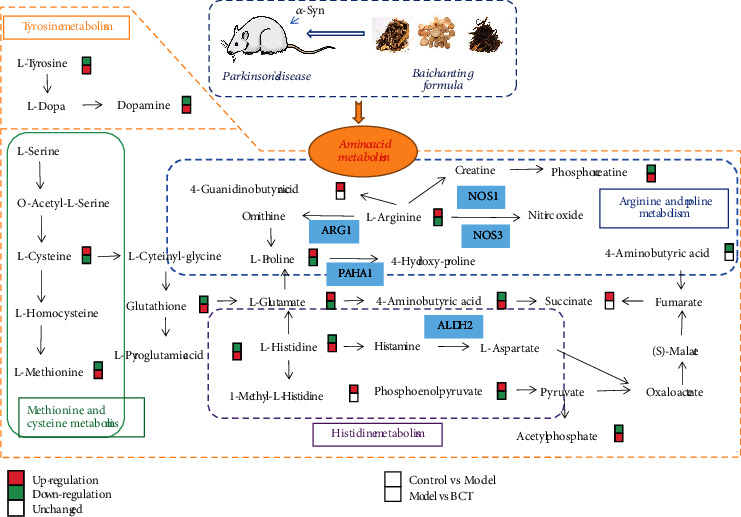
Schematic diagram of metabolic pathways.

Arginine is a semiessential amino acid that acts as a substrate for NO production by Nitric oxide synthase (NOS) and a precursor to a variety of metabolites, including ornithine, creatine, polyamines, and guanidine. Arginine mainly exists in the form of L-arginine (L-ARG), which is an important physiological role in nutrient metabolism and immune regulation. Arginine is established from arginine succinate synthase catalyzed by arginine succinate synthase. As an essential cellular communication signal, NO plays an important role in neurophysiology and neuroprotection, especially in neural survival and differentiation [29]. However, the effects of NO on the nervous system are two-sided, overactivating N-methyl-D-aspartate (NDMA) receptors, causing neuronal damage [30]. Excessive production of NO in NOS may lead to neuronal excitability toxicity and energy consumption, and is accompanied by the generation of ROS include nitrite peroxide and superoxide, these substances can cause oxidative stress injury of brain tissue, leading to a series of nerve pathological process, such as neurodegenerative diseases, inflammation, and cause cerebral ischemia reperfusion injury [30, 31]. As the final step in the urea cycle, arginase (AGR1) catalyzes l-arginine to form urea and L-ornithine. Arg1 is expressed primarily in neurons in the normal brain. Arg1 expression is increased in microglia/macrophages and astrocytes early after CNS injury [32]. Arg1 is considered a marker of beneficial microglia/macrophages and has anti-inflammatory and tissue repair properties in various pathological conditions [33]. Our experiments found that the content of Arg1 was significantly increased in PD, which may be due to the induction of NOS expression in the pathological state of brain tissue, resulting in excessive NO production. When Arg1 competes with inducible nitric oxide synthase (iNOS) for arginine substrates, can effectively downregulate NO production, thereby reducing the burden of intracellular oxidative stress during various cellular activities. In addition, L-ornithine synthesizes L-proline through the activity of ornithine aminotransferase. Proline is oxidized to pyrroline-5-carboxylic acid by proline oxidase (PRODH/POX). This transformation results in the formation of reactive oxygen species (ROS), which further trigger signaling cascades including processes such as autophagy and apoptosis [34]. Elevated levels of L-proline are associated with posttraumatic neurological impairment [35]. Extracellular matrix remodeling, especially collagen synthesis, requires the involvement of L-proline. Prolyl 4-hydroxylase subunit *α*1 (P4HA1) is the rate-limiting subunit of prolyl 4-hydroxylase (P4H), P4HA1 can regulate collagen synthesis and secretion in fibroblasts, thereby changing the composition of extracellular matrix [36]. P4HA1 is essential for posttranslational modification of collagen by catalyzing the formation of 4-hydroxyproline from proline residues [37]. Proline is a central inhibitory neurotransmitter. intracerebroventricular injection of proline can cause the decline of learning ability and the loss of retrograde memory in mice. Our experimental results found that the content of arginine and proline in the brain tissue of *α*-Syn transgenic mice increased, which may be caused by the inhibition of the decomposition of arginine after the occurrence of PD. The reduced breakdown of arginine results in lower levels of its metabolite, arginine succinate, which may lead to subsequent neurological damage, with adverse effects on the body. And BCT can reduce the content of arginine and proline, and increase the content of Phosphocreatine. qPCR results confirmed that the mRNA content of NOS1 and NOS3 decreased, suggesting that BCT may reduce NO production by inhibiting NOS activity, and partially block the signaling pathway of abnormal NO production by scavenging free radicals, thus reducing neuronal apoptosis (Figure 6).

4-aminobutyric acid (GABA) is considered as an important inhibitory neurotransmitter in the brain, which can reduce nerve excitability and protect the center [38]. GABA can be synthesized through argimime (Arg) and Spermine metabolism. It can also be catalyzed by *γ*-aminobutyric acid transaminase to generate succinic semialdehyde, and succinic semialdehyde dehydregenase (SSADH) can be converted into succinic acid and enter the tricarboxylic acid cycle again. As an intermediate product of the branch pathway of the tricarboxylic acid cycle, GABA is closely related to the energy cycle, and GABA plays a role as a regulator of oxidative metabolites. In addition, aldehyde dehydrogenase 2 (ALDH2), a key enzymatic protein in mitochondria, deletion leads to enhanced oxidative stress and neuronal death, and reduced ALDH2 activity is regarded as responsible for dopaminergic neurodegeneration in PD [39]. Toxic chemicals created by the metabolism of lipids and neurotransmitters build up in the central nervous system as a result of excessive oxidative stress. Chiu [40] discovered that ALDH2 mitigated rotenone-induced neuronal death in a rotenone-induced PD animal model, demonstrating the critical role that ALDH2 plays in maintaining normal mitochondrial activity to prevent neurotoxicity. These findings imply that neuroprotective effects of ALDH2 activation in Parkinson's disease. Our experimental results showed that GABA decreased in *α*-Syn transgenic mice, but the content of succinate increased, it is speculated that GABA deficiency, resulting in increased cholinesterase activity, accelerated hydrolysis of acetylcholine, decreased ALDH2 content, mitochondrial dysfunction, resulting in nerve impulse conduction disorders, affecting the normal function of the nerve. BCT can significantly increase the content of GABA, reduce blood ammonia, and promote the metabolism of brain cells. By oxidizing toxic compounds produced in the central nervous system into nontoxic metabolites, the function of brain cells can be restored. In addition, GABA can also be catalyzed by GLUT amic acid decarboxylase (GAD) to decarboxylase L-glutamic acid, which can antagonize the excitatory toxicity of glutamic acid. At the same time, the experiment also found that the content of GSH in the model group was reduced, and it was speculated that glutamate overactivation could stimulate the excitotoxic process and then participate in the pathogenesis of PD, resulting in the massive production of oxygen free radicals in brain tissues and the defects of oxygen free radical scavenging system, leading to the intracellular neurotoxic cascade reaction. The pharmacodynamic indexes MDA and SOD also verified that oxidative stress plays an important role in the regulation of cell function and is an important cause of neuronal degeneration in PD. BCT can increase the content of GSH, reduce the excessive accumulation of oxygen free radicals in the brain, resist the damage of dopaminergic neurons in the brain, and play a neuroprotective role.

**Figure 7:**
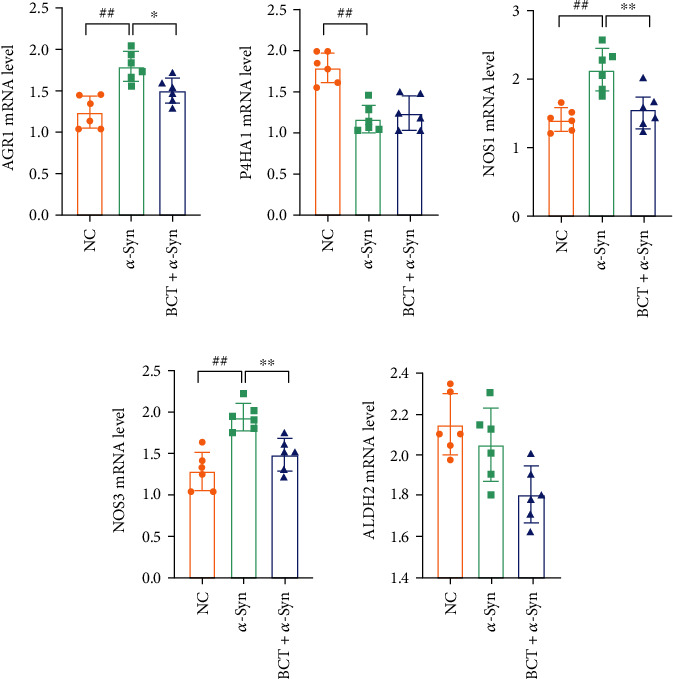
Quantification of expression levels of ARG1, P4HA1, NOS3, NOS1, and ALDH2 mRNA in three groups were measured using Real-time PCR. 2^−△△Ct^ method was used to analyze relative gene expression levels. Values expressed as mean ± SD (*n* = 6). ^#^ Significantly different from the control group at *P* < 0.05. ^##^ Significantly different from the control group at *P* < 0.01. ^∗^ Significantly different from the model group at *P* < 0.05. ^∗∗^ Significantly different from the model group at *P* < 0.01 (one-way ANCOVA).

Metabolomics, as an analytical technique to study the current metabolic status of the body, provides a new perspective for understanding the multifactorial mechanism of diseases and evaluating drug effects from a comprehensive and holistic perspective. At the same time, network pharmacology has great potential to reveal the mechanism of action of multicomponent drugs, forming complex interaction networks based on multicomponent, multitarget, and biological functions. It is of great significance for screening effective compounds, explaining the underlying mechanism of TCM, ensuring the quality of TCM, and rationally applying TCM. Our results further solidify the relationship between amino acid metabolism and PD. The integration of metabolomic and network pharmacology results showed that arginine metabolism and proline metabolism and histidine metabolism were closely related to the potential of BCT to improve PD. In our study, it was found that BCT compound showed multitarget effect as an anti-PD drug. First, it is demonstrated that BCT has properties of a number of different chemicals and a number of different targets. Second, pathway analysis showed that BCT might control several Parkinson's-related signaling pathways to have a combined effect. Target genes predicted by network pharmacology analysis (ARG1, P4HA1, NOS3, NOS1, and ALDH2) were associated with the regulation of potential differential metabolites (Figure 7). The results of qPCR showed that BCT can effectively regulate the mRNA levels of ARG1, P4HA1, NOS3, NOS1, and ALDH2 (Figure 7). It was further verified that BCT acts on these targets to play a role in the treatment of PD. According to the results of network pharmacology analysis, genes related to amino acid metabolism play a key role in mediating the anti-PD activity of BCT, and regulation of amino acid metabolism may be one of the main mechanisms of anti-PD effect of BCT combination. Therefore, combined brain metabolomics approaches the combined effect from two aspects. In addition, it is worth noting that arginine and proline metabolism and histidine metabolism are key related pathways based on integrated metabolomics and network pharmacology. And elucidated the metabolomics, multicompound, multitarget, and multimechanism of BCT anti-PD drugs. These findings help to reveal the combined mechanism of BCT against PD, and provide a reasonable way to clarify the composition of Traditional Chinese medicine.

## 4. Conclusion

On this basis, our experiment take the great idea of traditional Chinese medicine and modern science and technology of microcombined, in the view of traditional Chinese medicine as the guiding ideology, the potential effect of PD screening BCT therapeutic, target genes and key signaling pathways, analyze the differences between endogenous metabolites and metabolic pathways, and provide support to develop innovative Chinese medicine to treat PD. Compounds in BCT were subjected to network analysis, and 101 prospective target genes for PD therapy were used. In addition, BCT resistance to PD was strongly correlated with 25 different endogenous metabolites. These distinct metabolites participate in different amino acid metabolic pathways, and BCT may influence PD through controlling these processes. These findings matched those of the network pharmacologic analysis. The findings of this study demonstrate the necessity for other PD treatments to be suggested in the future. Currently, network pharmacology of traditional Chinese medicine is a hot topic in research. In the hunt for new PD therapy options, network pharmacology of TCM is currently a hot topic. A variety of models, target prediction, big data screening, and other cutting-edge techniques can be built through the analysis of the disease-gene-target-drug interaction network of the known active components and targets of TCM in order to achieve an overall, systematic, and thorough exploration of the effects of the mechanism of action of TCM on diseases. New approaches should also be developed to create alternative PD therapy. Future studies should examine if, in addition to amino acid metabolism, there are additional important pathways and targets involved in the development and therapy of PD. There are restrictions in how our results should be interpreted, despite the fact that we have clarified the therapeutic mechanism of BCT for PD using metabolic studies. To further represent the mechanism by which BCT affects PD, global metabolic changes in additional structures (such as the hippocampus and cerebrospinal fluid) should be identified. For the deliberate detection of metabolite biomarkers, advanced metabolomic technologies, such as targeted metabolomics, would also be required. This is an issue for future research to explore. In summary, this paper will provide data support and theoretical support for in-depth study of the mechanism of BCT in the treatment of PD, thus laying a foundation for further clinical applications in the future.

## Figures and Tables

**Table 1:** Gene primers used for real-time PCR.

Gene	Forward primer	Reverse primer
GAPDH	5′-TTTGAGGGTGCAGCGAACTT-3′	5′-ACAGCAACAGGGTGGTGGAC-3′
ARG1	5′-CTTGCGAGACGTAGACCCT-3′	5′-AATCGGCCTTTTCTTCCTTCC-3′
P4HA1	5′-ACCACAGCACAGTACAGAGTAT-3′	5′-GGAAACATCCAGTCCTGTGAG-3′
NOS3	5′-CTGACAGCTCCTGTTCGGGT-3′	5′-GCGCAATGTGAGTCCGAAAA-3′
NOS1	5′-CTGGTGAAGGAACGGGTCAG-3′	5′-CCGATCATTGACGGCGAGAAT-3′
ALDH2	5′-CAGATGACTGCCCAACTCCC-3′	5′-TGAACCCAGGTCCTCGCTTA-3′

**Table 2:** The identifification of potential biomarkers.

No.	ESI mode	Metabolites	VIP	Measured mass(Da)	Calculated mass (Da)	Error m/z (ppm)	RT (s)	Control vs model trend	Model vs PT trend
1	Neg	Phosphocreatine	1.5024	211.033	211.1131	0.0801	362.592	↓	↑
2	Neg	Galactonic acid	1.5926	196.051	196.1553	0.1043	371.962	↓	↑
3	Neg	Succinate	1.4109	118.019	118.088	0.069	387.540	↑	↓
4	Neg	Acetyl phosphate	1.0908	139.936	140.0319	0.0959	499.938	↓	↑
5	Neg	L-glutamate	1.1974	147.046	147.1293	0.0833	396.310	↑	↓
6	Neg	L-histidine	1.0672	155.062	155.1546	0.0926	425.167	↓	↑
7	Neg	L-methionine	1.0852	149.043	149.211	0.168	310.026	↓	↑
8	Neg	N-acetylmannosamine	1.4454	221.104	221.2078	0.1038	356.177	↓	↑
9	Neg	Nervonic acid	1.1663	366.342	366.6208	0.2788	125.617	↑	↓
10	Neg	Phosphoenolpyruvate	1.0987	167.964	168.042	0.078	458.221	↑	↓
11	Neg	Prostaglandin E2	1.0286	352.218	352.4651	0.2471	91.224	↑	↓
12	Neg	Taurocholate	1.0506	515.285	515.703	0.418	198.837	↓	↑
13	Neg	L-tyrosine	1.1096	181.066	181.1885	0.1225	302.726	↓	↑
14	Pos	1-Methylhistidine	1.6921	169.091	169.1811	0.0901	352.570	↑	↑
15	Pos	Alpha-linolenic acid	1.0387	279.257	278.4296	-0.8274	33.231	↓	↑
16	Pos	Argininosuccinic acid	1.2715	290.130	290.2731	0.1431	459.555	↓	↑
17	Pos	Dopamine	1.0788	153.075	153.1784	0.1034	291.703	↓	↑
18	Pos	Glutathione	1.6425	307.090	307.32	0.23	448.013	↓	↓
19	Pos	L-arginine	2.2639	174.118	174.201	0.083	393.471	↑	↓
20	Pos	L-cysteine	1.9694	122.051	121.158	-0.893	227.686	↑	↓
21	Pos	L-proline	1.4740	115.070	115.1305	0.0605	300.404	↑	↑
22	Pos	L-pyroglutamic acid	1.2330	130.075	129.114	-0.961	392.084	↓	↑
23	Pos	Prostaglandin I2	1.2633	352.230	352.4651	0.2351	202.732	↓	↑
24	Pos	4-Guanidinobutyric acid	1.4416	145.091	145.1597	0.0687	349.299	↑	↓
25	Pos	4-Aminobutyric acid	1.9662	103.069	103.1198	0.0508	362.801	↓	↑

**Table 3:** Target information of BCT treating PD in network pharmacology.

No.	Gene symbol	Uniprot ID	Protein name
1	ALDH2	P05091	Aldehyde dehydrogenase
2	LAP3	P28838	Cytosol aminopeptidase
3	P4HA1	P13674	Prolyl 4-hydroxylase subunit alpha-1
4	NOS3	P29474	Nitric oxide synthase
5	AMD1	P17707	S-adenosylmethionine decarboxylase proenzyme
6	NOS1	P29475	Nitric oxide synthase
7	GSR	P00390	Glutathione reductase
8	TP53	P04637	Cellular tumor antigen p53
9	SLC6A3	Q01959	Sodium-dependent dopamine transporter
10	LRRK2	Q5S007	Leucine-rich repeat serine/threonine-protein kinase 2
11	BCL2L1	Q07817	Bcl-2-like protein 1
12	MAPT	P10636	Microtubule-associated protein tau
13	MAP3K5	Q99683	Mitogen-activated protein kinase kinase kinase 5
14	PSMB5	P28074	Proteasome subunit beta type-5
15	DRD1	P21728	D(1A) dopamine receptor (dopamine D1 receptor)
16	MAPK8	P45983	Mitogen-activated protein kinase 8
17	MAPK9	P45984	Mitogen-activated protein kinase 9
18	GLUL	P15104	Glutamine synthetase
19	GAPDH	P04406	Glyceraldehyde-3-phosphate dehydrogenase
20	ARG1	P05089	Arginase-1
21	IDH1	O75874	Isocitrate dehydrogenase [NADP] cytoplasmic
22	TLR4	O00206	Toll-like receptor 4
23	TNF	P01375	Tumor necrosis factor
24	AOC3	Q16853	Membrane primary amine oxidase
25	SPTLC1	O15269	Serine palmitoyltransferase 1
26	GLB1	P16278	Beta-galactosidase
27	GBA	P04062	Lysosomal acid glucosylceramidase
28	GBA2	Q9HCG7	Non-lysosomal glucosylceramidase
29	FBP1	P09467	Fructose-1
30	PLA2G1B	P04054	Phospholipase A2
31	CYP3A4	P08684	Cytochrome P450 3A4
32	HK2	P52789	Hexokinase-2
33	HSPA5	P11021	Endoplasmic reticulum chaperone BiP
34	DAO	P14920	D-amino-acid oxidase
35	GAA	P10253	Lysosomal alpha-glucosidase
36	NOX4	Q9NPH5	NADPH oxidase 4
37	MAPK10	P53779	Mitogen-activated protein kinase 10
38	CXCR2	P25025	C-X-C chemokine receptor type 2
39	ERN1	O75460	Endoplasmic reticulum-to-nucleus signaling 1
40	CASP3	P42574	Caspase-3
41	PTGS2	P35354	Prostaglandin G/H synthase 2
42	HCAR2	Q8TDS4	Hydroxycarboxylic acid receptor 2
43	TK1	P04183	Thymidine kinase
44	MAP3K7	O43318	Mitogen-activated protein kinase kinase kinase 7
45	SLC6A4	P31645	Sodium-dependent serotonin transporter
46	RAF1	P04049	RAF proto-oncogene serine/threonine-protein kinase
47	DRD4	P21917	Dopamine D4 receptor
48	GSK3A	P49840	Glycogen synthase kinase-3 alpha
49	MAPK14	Q16539	Mitogen-activated protein kinase 14
50	PRKCG	P05129	Protein kinase C gamma type
51	GRIA2	P42262	Glutamate receptor 2
52	PRKCB	P05771	Protein kinase C beta type
53	DRD5	P21918	Dopamine D5 receptor
54	GSK3B	P49841	Glycogen synthase kinase-3 beta
55	MAPK11	Q15759	Mitogen-activated protein kinase 11
56	DRD2	P14416	Dopamine D2 receptor
57	DRD3	P35462	Dopamine D3 receptor
58	PRKCA	P17252	Protein kinase C alpha type
59	PPP2CA	P67775	Serine/threonine-protein phosphatase 2A catalytic subunit alpha isoform
60	AKT1	P31749	RAC-alpha serine/threonine-protein kinase
61	LYN	P07948	Tyrosine-protein kinase Lyn
62	ALOX12	P18054	Polyunsaturated fatty acid lipoxygenase ALOX12
63	CYP2C9	P11712	Cytochrome P450 2C9
64	PTGES	O14684	Prostaglandin E synthase
65	PTGS1	P23219	Prostaglandin G/H synthase 1
66	CYP2C19	P33261	Cytochrome P450 2C19
67	ALOX5	P09917	Polyunsaturated fatty acid 5-lipoxygenase
68	EPHX2	P34913	Bifunctional epoxide hydrolase 2
69	OPRD1	P41143	Delta-type opioid receptor
70	CTSD	P07339	Cathepsin D
71	MAP2K2	P36507	Dual specificity mitogen-activated protein kinase kinase 2
72	MAPK3	P27361	Mitogen-activated protein kinase 3
73	PLD2	O14939	Phospholipase D2
74	AKT3	Q9Y243	RAC-gamma serine/threonine-protein kinase
75	PIK3CA	P42336	Phosphatidylinositol 4
76	KNG1	P01042	Kininogen-1
77	MAP2K1	Q02750	Dual specificity mitogen-activated protein kinase kinase 1
78	ROCK2	O75116	Rho-associated protein kinase 2
79	PLD1	Q13393	Phospholipase D1
80	FYN	P06241	Tyrosine-protein kinase Fyn
81	ADORA1	P30542	Adenosine receptor A1
82	ROCK1	Q13464	Rho-associated protein kinase 1
83	HRAS	P01112	GTPase HRas
84	MAPK1	P28482	Mitogen-activated protein kinase 1
85	HTR2B	P41595	5-hydroxytryptamine receptor 2B
86	HTR2C	P28335	5-hydroxytryptamine receptor 2C
87	BRAF	P15056	Serine/threonine-protein kinase B-raf
88	HTR6	P50406	5-hydroxytryptamine receptor 6
89	HTR1A	P08908	5-hydroxytryptamine receptor 1A
90	HTR7	P34969	5-hydroxytryptamine receptor 7
91	CYP2D6	P10635	Cytochrome P450 2D6
92	PLA2G4A	P47712	Cytosolic phospholipase A2
93	HTR1B	P28222	5-hydroxytryptamine receptor 1B
94	HTR2A	P28223	5-hydroxytryptamine receptor 2A
95	GRM5	P41594	Metabotropic glutamate receptor 5
96	ADRBK1	P25098	Beta-adrenergic receptor kinase 1
97	GLS	O94925	Glutaminase kidney isoform
98	GRM1	Q13255	Metabotropic glutamate receptor 1
99	GRIK1	P39086	Glutamate receptor ionotropic
100	MAOA	P21397	Amine oxidase [flavin-containing] A
101	MAOB	P27338	Amine oxidase [flavin-containing] B

**Table 4:** The information of key targets, metabolites, and pathways.

Related pathway	Key target	Key metabolite
Arginine and proline metabolism	ARG1, P4HA1, NOS3, NOS1, and ALDH2	L-arginine, L-proline, and 4-aminobutyric acid, Argininosuccinic acid
Histidine metabolism	ALDH2	L-histidine and 1-Methylhistidine

## Data Availability

The data that support the findings of this study are available from the corresponding author, upon reasonable request.

## Abbreviations

BCT: Baichanting
PD: Parkinson's disease
TCM: Traditional Chinese medicine
HMDB: Human metabolome database
PPI: Protein-protein interaction
GSH: Glutathione
Glu: Glutamate
SOD: Superoxide dismutase
MDA: Malondialdehyde
PCA: Principal component analysis
PLS-DA: Partial least squares discriminant analysis
KEGG: Kyoto encyclopedia of genes and genomes
TCA: Tricarboxylic acid
NO: Nitric oxide
NOS: Nitric oxide synthase
DA: Dopamine
AGR1: Arginase
P4HA1: Prolyl 4-hydroxylase subunit *α*1
GABA: 4-Aminobutyric acid
ALDH2: Aldehyde dehydrogenase 2.
